# Trophic Tangles through Time? Opposing Direct and Indirect Effects of an Invasive Omnivore on Stream Ecosystem Processes

**DOI:** 10.1371/journal.pone.0050687

**Published:** 2012-11-27

**Authors:** Jonathan W. Moore, Stephanie M. Carlson, Laura A. Twardochleb, Jason L. Hwan, Justin M. Fox, Sean A. Hayes

**Affiliations:** 1 Department of Ecology and Evolutionary Biology, University of California Santa Cruz, Santa Cruz, California, United States of America; 2 Department of Environmental Science, Policy, and Management, University of California, Berkeley, California, United States of America; 3 Southwest Fisheries Science Center, National Oceanic and Atmospheric Administration Fisheries Service, Santa Cruz, California, United States of America; National Institute of Water & Atmospheric Research, New Zealand

## Abstract

Omnivores can impact ecosystems via opposing direct or indirect effects. For example, omnivores that feed on herbivores and plants could either increase plant biomass due to the removal of herbivores or decrease plant biomass due to direct consumption. Thus, empirical quantification of the relative importance of direct and indirect impacts of omnivores is needed, especially the impacts of invasive omnivores. Here we investigated how an invasive omnivore (signal crayfish, *Pacifastacus leniusculus*) impacts stream ecosystems. First, we performed a large-scale experiment to examine the short-term (three month) direct and indirect impacts of crayfish on a stream food web. Second, we performed a comparative study of un-invaded areas and areas invaded 90 years ago to examine whether patterns from the experiment scaled up to longer time frames. In the experiment, crayfish increased leaf litter breakdown rate, decreased the abundance and biomass of other benthic invertebrates, and increased algal production. Thus, crayfish controlled detritus via direct consumption and likely drove a trophic cascade through predation on grazers. Consistent with the experiment, the comparative study also found that benthic invertebrate biomass decreased with crayfish. However, contrary to the experiment, crayfish presence was not significantly associated with higher leaf litter breakdown in the comparative study. We posit that during invasion, generalist crayfish replace the more specialized native detritivores (caddisflies), thereby leading to little long-term change in net detrital breakdown. A feeding experiment revealed that these native detritivores and the crayfish were both effective consumers of detritus. Thus, the impacts of omnivores represent a temporally-shifting interplay between direct and indirect effects that can control basal resources.

## Introduction

The impacts of omnivores on food webs and ecosystems remain difficult to predict. Many, if not most, species feed on multiple prey and at multiple trophic levels [1]–[5]. Such trophic omnivores can impact basal resources such as detritus or plants through opposing direct (e.g., consumption) and indirect (e.g., trophic cascade) pathways. Thus, the addition of an omnivorous consumer that consumes plants as well as herbivores may lead to an increase, a decrease, or no net change in plant biomass (e.g., [6]–[9]). Our understanding of the impacts of such omnivores lags behind our understanding of the impacts of top consumers. Classic papers [10]–[12] argued that the addition of a top predator will drive a trophic cascade, leading to predictable changes in plant biomass. Regardless of whether trophic cascades are mediated by changes in prey density or traits, there are now examples of such trophic cascades in a variety of ecosystems [13]–[15], including rocky intertidal [11], kelp forest [16], lake [17], stream [18], and terrestrial systems [19]. There is also evidence of cascading impacts of top predators on the detrital base of food webs [20], [21]. Time lags in the propagation of indirect effects may further complicate understanding of the impacts of consumers [22]–[24]. Thus, predicting the impacts of omnivores remains a challenge.

Quantifying the impacts of omnivores is particularly important for invasive species [25]. In many regions, invasive species are now major components of communities and contribute substantially to ecosystem processes, such as water filtration [26]. In addition, the invasion of a single species can rapidly alter communities and ecosystem processes [27]. Given that the impacts of omnivores are difficult to predict, it follows that invasive omnivores may drive ‘ecological surprises’ (sensu [28]).

We focused this study on an invasive omnivore, signal crayfish (*Pacifastacus leniusculus*), an ideal study species to examine the interplay between potentially opposing direct and indirect effects. Crayfish are consummate generalists; for example, they feed on primary production such as filamentous algae, detritus such as leaf litter, as well as other benthic invertebrates [29], [30]. Thus, crayfish could exert direct negative effects on algal biomass via grazing [31]–[34] or indirect positive effects via a trophic cascade by feeding on herbivorous invertebrates that consume vulnerable algae [35], [36]. Alternatively, crayfish can directly consume detritus such as terrestrial leaf litter but could also reduce detritus processing by their consumption or disruption of detritivorous invertebrates [30], [37]. Understanding the impacts of crayfish is particularly relevant because they are important invasive species as well as imperiled native species in different regions [35], [38].

We investigated the impacts of an invasive omnivorous consumer in a stream food web. We conducted our study in headwater streams where autochthonous (e.g., benthic algae) sources of energy are reduced due to shading while allochthonous (e.g., terrestrial leaf litter detritus) pathways are commonly the dominant source of energy and nutrients [39]. We used a combination of a large-scale experiment, a comparative study, and feeding trials to address the questions: Do crayfish have net negative or net positive impacts on stream invertebrates, algae, and leaf litter processing rates? Do the effects of crayfish depend on temporal scale? Specifically, we were interested in how the patterns of direct and indirect effects of crayfish differed between the short-term experiment and the comparative approach. We predicted that crayfish would have direct effects on benthic invertebrates, algal accrual, and leaf litter breakdown via consumption, and opposing indirect effects on algal accrual and leaf litter breakdown via predation on other benthic invertebrates. Additionally, we predicted that direct effects would dominate the short-term experiment, whereas indirect effects would take primacy in the long-term comparative study.

## Materials and Methods

### Study Sites and Systems

This study was performed in Scott Creek, a small coastal watershed in central California (37°3’43.53″N, 122°13’42.53″W). The upper portions of this watershed are high-gradient stream channel and forested; riparian areas generally have an over-story of coastal redwoods (*Sequoia sempervirens*) and an understory of red alder (*Alnus rubra*). This watershed also has two large waterfalls in the two largest tributaries–Big Creek and Upper Scott Creek. Signal crayfish are the only crayfish species in the watershed. They are native to primarily Oregon, Washington, and northern California of the United States and British Columbia of Canada [38], [40], [41] but are nonnative to central and southern California. Anecdotal evidence suggests that they invaded the study watershed roughly 90 years ago. Based on conversations with members of the McCrary family, whom have lived in the watershed since 1869 and collected detailed natural history notes, signal crayfish were introduced to the watershed in the 1920s in the upper reaches of the tributary Big Creek, upstream of a ∼40 m high waterfall. From this upstream origin, it is thought that they spread downstream and then dispersed throughout the watershed. However, a ∼20 m waterfall in Upper Scott Creek apparently blocked their spread, as they are not found upstream of this migration barrier. This migration barrier allowed a comparison of invaded and un-invaded areas (see Comparative study).

### Experimental Density Manipulation

During the summer of 2008, we performed a large-scale density-manipulation field experiment to examine how crayfish impact stream ecosystems over a three month period (July – October, 75 days). We used isolated stream pools during the summer low flow period as units of replication. During the low flows of summer, pools become isolated as water percolates through the gravel of riffles. Thus, these pools offer natural units of replication in which to study community dynamics. We selected 16 study pools (area = 69.8±26.7 m^2^, this and the following are average ±1 standard deviation) from a 2 km reach of Upper Scott Creek. Pools were randomly assigned to crayfish removal or addition within four blocks to ensure spatial spread of the density manipulation. We entrenched a fence of 0.5 cm diameter mesh netting at the top and bottom of each study pool to further minimize movement in and out of each study pool. No substantial rain occurred during the experiment, and stream water flows were consistent.

The crayfish density manipulation was initiated via a three-pass backpack electro-fishing at the beginning of the experiment, where captured vertebrates and crayfish were measured and weighed in every study pool. Pre-manipulation crayfish densities averaged 0.45±0.22 individuals m^−2^ with an average biomass density of 4.09±2.86 g m^−2^. Captured crayfish were removed from “removal” pools, and were added to the “addition” pools to reach a target density that was on the upper end of observed natural densities. Specifically, the target density for the “addition” pools was 0.74 individuals m^−2^ equating to a biomass of 6.3 g m^−2^. Crayfish densities were maintained every 10–14 days using unbaited crayfish traps and hand-netting, and moving crayfish among pools when necessary. Crayfish losses or gains were kept relatively constant by adding or reducing crayfish in pools in order to maintain a relatively continuous gradient of crayfish densities. Thus, hereafter crayfish density is treated as a continuous variable.

Stream reaches also contained other vertebrates. All other captured vertebrates were returned to the pool that they were caught from. These other species included rainbow trout (*Oncorhynchus mykiss*), three-spine stickleback (*Gasterosteus aculeatus*), and prickly sculpin (*Cottus asper*). The dominant (by numbers and biomass) fish species were rainbow trout. Rainbow trout predominantly feed on invertebrates in the drift, contrasting to crayfish that feed on benthic habitat. As noted below, densities of these other consumers did not correlate significantly with crayfish densities, assuaging concerns that other consumers could be driving the observed patterns.

We estimated fish and crayfish abundance with snorkeling surveys and separate electro-fishing depletion. We used standard three-pass electro-fishing depletion [42] at the beginning and end of the experiment. This method of population estimation uses the sequential depletion of individuals to estimate the total population size [42]. In some cases, three-pass depletion population estimates were unrealistic due to low sample sizes in smaller pools; in these cases we estimated abundance as total observed individuals multiplied by the average ratio of total observed: total estimated seen across the other pools. We also quantified crayfish densities with a snorkeling survey during the day performed half-way through the experiment. Numerical abundance was converted to biomass density by multiplying abundance by average mass of an individual from each pool and dividing by pool surface area. While density estimates were correlated (*r* = 0.54) for these two time periods, crayfish densities were somewhat dynamic over the course of the experiment due to natural processes such as rare movement of individuals, as evidenced by recapture of two individually-marked crayfish that had moved to different pools, and predation by raccoons, as suggested by footage from motion sensor cameras. Therefore, we averaged the density estimate from the middle of the experiment with the density estimate from the end of the experiment. This average density estimate thereby represents the best estimate of average crayfish density in a given pool over the course of the experimental period. Crayfish density manipulation generated a gradient of crayfish densities that was not significantly related with environmental variables such as light (*r* = 0.11, *P* = 0.69) or degree-days (*r* = 0.20, *P* = 0.57) nor the densities of other consumers such as rainbow trout (*r* = 0.24, *P* = 0.38) and sculpin (*r* = 0.16, *P* = 0.54).

We quantified the benthic invertebrate community at the end of the experiment through use of a Surber sampler (0.1 m^2^ in area, mesh size = 500 µm) from the middle of each pool. Invertebrates were sorted to family and up to 20 individuals from each family were measured for total length. These lengths were converted to mass estimates using length-weight regressions [43]. We also collected organic matter via Surber sampler–all detritus was dislodged from the Surber area into the downstream net. We quantified organic matter by collecting the dry mass before and after the sample was combusted at 550 degrees C [44]. At the beginning of the experiment, unglazed ceramic tiles were placed in each pool, and sequentially recovered every two to three weeks to quantify the rate of algal accrual. This sampling regime favors small algae that grow rapidly such as diatoms. We quantified algal accrual rates on tiles that were either placed directly on the substrate (hereafter “control tiles”) or on an elevated platform 20 cm above the substrate with rebar stakes that acted to reduce potential herbivory by invertebrates that do not swim or drift (hereafter “elevated tiles”) [44]. This elevated platform could have also influenced other factors that could influence the growth of algae, including water flow, and fine sediment deposition. Thus, we are considering these two different tile locations as a way to investigate how crayfish influence algal accrual in two different types of conditions with potentially different grazer communities. Algal biomass was quantified by retrieving tiles, scrubbing them with a small brush, and filtering a known fraction of the filtrate through 47 mm GF/F silica filters (pore size 0.7 µm). Filters were frozen and later solubilized in methanol and the concentration of chlorophyll-a was determined with fluorometry [44]. Algal accrual rates were estimated as the slope of the linear relationship between date and algal biomass, estimated 2–4 times over the course of the study. Data inspection indicated that a linear relationship consistently fit better than an exponential relationship. We quantified pool-specific leaf litter breakdown rates using coarse mesh bags (1 cm mesh size) containing pre-weighed dry red alder leaves that were sequentially recovered every two to three weeks (recovered two each recovery period with a total of 3 collection periods). This mesh size is large enough for crayfish and other benthic invertebrates to access the leaf litter, either directly by entering the bag, or for the larger crayfish to access the leaf litter with their claws. We frequently observed crayfish of a variety of sizes feeding on the leaf material in the leaf bags. The breakdown rate of leaves (k) was estimated according to an exponential decay model [44]. In each pool, we placed a waterproof Onset HOBO Temperature/Light Pendant Data Logger that collected hourly readings of stream temperature and light intensity. We also collected water nutrients during the experiment to examine the possibility that crayfish could be altering nutrient cycling, such as through excretion. We collected nutrients from each pool three times during the experiment. Water samples for nutrients were collected in acid-washed polyethylene bottles, filtered through a pre-ashed 47 mm GF/F silica filter (pore size = 0.7 µm), and frozen for subsequent analyses. Nitrite, nitrate, phosphate, and ammonium concentrations were quantified at the University of California Santa Barbara with a QuikChem 8000 flow injection analyzer.

### Comparative Study

We used a comparative field study to see if the results from the experiment scaled up in time. During the summer of 2009, we compared pools that spanned a natural gradient in crayfish densities (range 0–6.74 g m^−2^, mean = 1.72 g m^−2^). In order to include sites without crayfish, we focused on pools that were above and below a ∼20 m barrier waterfall in Upper Scott Creek. No crayfish are found above this migration barrier. Rainbow trout (*Oncorhynchus mykiss*), the major vertebrate predator, were present in all study reaches, both above and below waterfalls. We compared four pools without crayfish above this barrier waterfall to eight pools throughout the watershed that contained crayfish. Specifically, the eight pools with crayfish consisted of: two pools downstream of the waterfalls in Upper Scott Creek, two pools downstream of the waterfalls on Big Creek, and four pools upstream of the waterfalls on Big Creek (the putative source of the crayfish invasion). This sampling was used in order to have sites that were a balance of above and below waterfalls, thereby incorporating potential effects of the waterfalls as dispersal barriers. Pool area averaged 45.9±19.88 m^2^ (±1 SD). Pool area was not significantly correlated with crayfish density (*P*>0.05, *R^2^* = 0.025). Following the same protocol as for the experimental study, we quantified light, temperature, algal accrual rate, and leaf litter breakdown rate. We snorkeled the pools during the day and counted crayfish during the study period. These counts were converted to biomass density by using average crayfish mass from the 2008 study and by dividing estimated total crayfish biomass by the measured surface area of the pool. We used the coarse mesh litter bags from the first round of our sampling to collect benthic invertebrates. A fire burned roughly half of our study pools during August 13–18, 2009, thereby truncating this study after 40 days. We only used samples collected before the fire. We note that the summers of 2008 and 2009 were climatically similar, with no precipitation from early July to the end of September. We examined how crayfish density, temperature (cumulative degree days), and light (cumulative light flux) influenced the response variables (see Statistical analysis below). We note that crayfish density was not significantly related to temperature (*r* = 0.14, *P* = 0.87) nor light (*r* = 0.17, *P* = 0.59).

### Feeding Trials

In order to gain further insight into patterns of leaf litter breakdown rates, we investigated the relative consumption rates of leaf litter by key benthic invertebrates during early August of 2009. Specifically, we performed feeding trials on three of the dominant shredders–the signal crayfish (*P. leniusculus*) and two large native caddisflies (order Tricoptera), *Lepidostoma* spp. and *Psychoglypha* spp. We had four groups of invertebrates (with four replicates of each): large and small size classes of crayfish and the two native caddisflies. We also had four control containers that had leaves but no added invertebrates to quantify background (microbial, physical) breakdown rate of leaves. We placed individual invertebrates in flow-through containers as well as a pre-weighed, pre-dried piece of alder leaf. Flow-through containers consisted of small plastic containers (approximately 500 ml volume) that had holes covered with mesh (mesh size 100 µm). Containers were placed in the study streams with the leaf material for 18 days for a conditioning period. The invertebrates and remaining leaf were removed after four to eight days and both were dried and weighed. We quantified consumption rate as the rate of mass loss of leaves (mg d^−1^), after having adjusted for mass loss in control containers. We anticipated that consumption would follow an allometric scaling relationship [45], and thus we fit a basic allometric scaling relationship (consumption = a*invertebrate mass^b^; where *b* is the power scaling parameter) using the nls function in R [46].

### Statistical Analyses

For both the comparative study and the experiment, we examined how response variables were influenced by crayfish density and key environmental variables, temperature (cumulative degree days) and light (cumulative light flux). To compare the relative strength of these predictor variables, we used a model comparison approach [47]. This approach allowed us to compare the relative support for crayfish driving the observed patterns versus environmental variables. We used AICc (Akaike Information Criterion corrected for small sample sizes) as the metric of model support [47]. We calculated ΔAICc as the difference in AICc between the model and the best model. A lower ΔAICc score indicates a model that is more supported by the data, with differences between values of at least 2 indicating substantially less support [47]. All analyses were implemented in R [46]. When we graphed the relationships between crayfish and response variables, we used the predict function of R to generate 95% confidence intervals. When results from the experiment were used to compare to the comparative study, we used prediction intervals using the predict function in R.

### Ethics

This study was carried out in accordance with ethical guidelines. The field work was performed on private land owned by the Big Creek Lumber Company, Swanton Pacific Ranch of California Polytechnic State University, and Lockheed Martin. All land use was approved by these land owners. Research on animals was approved for by the University of California Institutional Animal Care & Use Committee (Moorj0804), the California Department of Fish and Game (SC-10153), and National Marine Fisheries Service (1112, modification 2).

## Results

### Experimental Manipulation

**Figure 1:**
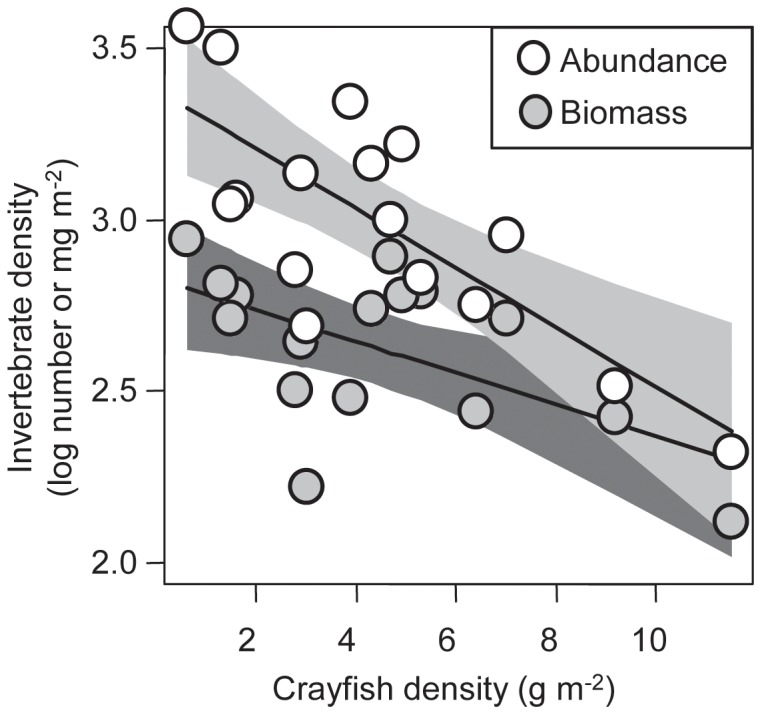
Results from the experimental manipulation of crayfish densities. Each point represents a study pool. Total non-crayfish benthic invertebrate abundance corresponds to open white symbols and gray confidence interval while total non-crayfish biomass corresponds to the gray symbols and darker gray confidence intervals. Benthic invertebrates were negatively associated with crayfish for both non-crayfish benthic invertebrate numerical density (log (invertebrates m^−2^) = −0.087 * crayfish +3.38, *R^2^* = 0.59, *P*<0.001) and biomass density (log (invertebrates m^−2^) = −0.046 * crayfish +2.82; *R^2^* = 0.33, *P* = 0.02). The solid lines denote these best fit linear model and the polygons indicate 95% confidence intervals. Note data are log-transformed.

Large-scale experimental manipulations of crayfish densities revealed widespread impacts of crayfish on stream benthic invertebrate communities on a rapid (3 months) timeframe. Total non-crayfish invertebrate abundance was significantly and negatively related to crayfish density (log (invertebrates m^−2^) = −0.087 * crayfish +3.38, *R^2^* = 0.59, *P*<0.001; Fig. 1). In addition, total non-crayfish invertebrate mass was also significantly and negatively related to crayfish density (log (invertebrates m^−2^) = −0.046 * crayfish +2.82; *R^2^* = 0.33, *P* = 0.02; Fig. 1). Non-crayfish invertebrate abundance was 10 times higher in pools with lowest crayfish densities than those with highest crayfish densities. Crayfish density was a substantially better model predictor of invertebrate abundance and biomass than either light or temperature, as indicated by ΔAICc scores (Electronic supplemental materials Table S1).

Post-hoc regressions between crayfish densities and taxa biomass revealed that different taxa appeared to be differently impacted by crayfish (see Fig. S1 in the Electronic supplemental material). Specifically, crayfish density was negatively associated with the biomass of Chironomidae (*R^2^* = 0.70, *P*<0.0001), Odonata (*R^2^* = 0.25, *P* = 0.046), and Tricoptera (*R^2^* = 0.25, *P* = 0.05). In contrast, other taxa were not significantly related to crayfish density, including Ephemeroptera (*R^2^* = 0.078, *P* = 0.293), Plecoptera (*R^2^* = 0.00, *P* = 0.98), and the biomass of an aggregated group of more uncommon taxa (predominantly Odonata, Dipterans from the family Tipulidae, Coleoptera, and Sialidae; *R^2^* = 0.07, *P* = 0.34). However, the average individual mass of invertebrates did not significantly vary across crayfish density for all taxa (*P*>0.1 for all taxa, results not shown).

**Figure 2:**
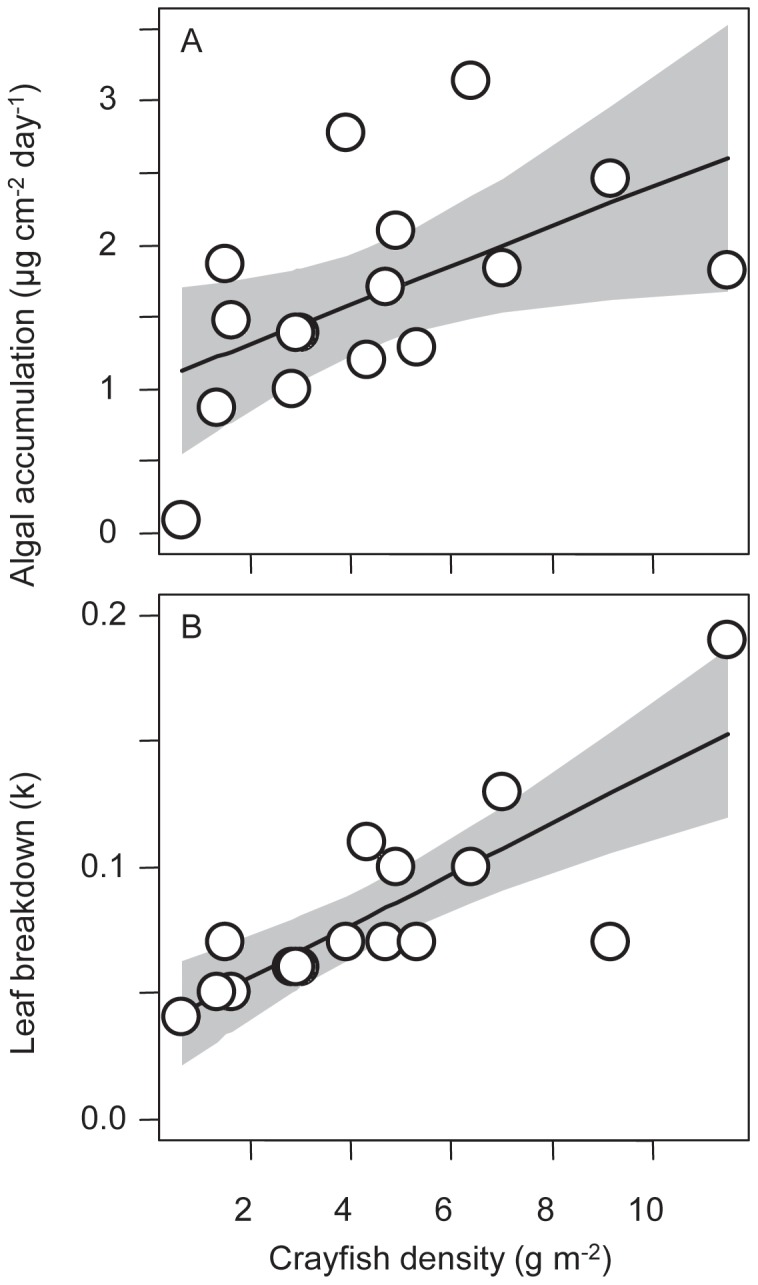
Ecosystem processes and crayfish density manipulation. Each point represents a study pool. (a). Rate of algal accrual on elevated tiles. Algal accrual was negatively associated with crayfish density (algal accrual rate = 0.13 * crayfish +1.05; *R^2^* = 0.29, *P* = 0.03). (b). Rate of breakdown of leaves. A higher k value corresponds to more rapid breakdown and k was higher at higher crayfish densities (k = 0.010 * crayfish +0.036; *R^2^* = 0.64, *P* = 0.0002). For both relationships, the solid line denotes the best fit linear model and the gray polygon indicates the 95% confidence interval.

Algal accrual on elevated tiles was significantly and positively related to crayfish density (algal accrual rate = 0.13 * crayfish +1.05; *R^2^* = 0.29, *P* = 0.03; Fig. 2a). Crayfish was a substantially better predictor of algal accrual on elevated tiles than either light (ΔAICc = 14.3) or temperature (ΔAICc = 11.6; Electronic supplemental materials Table S1). This pattern matches the predictions based on the indirect effects of a classic trophic cascade–i.e., crayfish predation on grazing benthic insects indirectly increased algal accrual. However, there was no significant relationship between crayfish density and accrual on control tiles (*R^2^* = 0.078; *P* = 0.29). Model comparison revealed that neither temperature nor light were substantially better models, all factors explained little of the observed variation in algal accrual on the control tiles (Electronic supplemental material Table S1).

Leaf litter breakdown was significantly and positively related to crayfish density (k = 0.010 * crayfish +0.036; *R^2^* = 0.64, *P* = 0.0002; Fig. 2b). Temperature (ΔAICc = 16.4) and light models (ΔAICc = 16.0) had essentially no support. We frequently observed crayfish directly consuming leaf litter, including in our mesh bags. Correspondingly, total benthic organic matter was lower when crayfish densities were higher (log (g organic matter m^−2^) = −0.087 * crayfish +1.969; *R^2^* = 0.32, *P* = 0.02); pools with high crayfish densities had an order of magnitude less organic matter than pools with low crayfish densities (0.94 vs. 10.46 g organic matter m^−2^). Crayfish was a considerably better predictor of carbon standing stock than either temperature (ΔAICc = 4.6) or light (ΔAICc = 5.5) (Electronic supplemental material Table S1).

Concentrations of dissolved nutrients were not significantly related to crayfish density (Electronic supplemental material Table S1). Specifically, crayfish density was not significantly related with the concentration of nitrite plus nitrate (µM of nitrate plus nitrite = 6.87 * crayfish +0.182; *R^2^* = 0.083, *P* = 0.28), phosphate (µM of phosphate = 0.002 * crayfish +0.61, *R^2^* = 0.003, *P* = 0.85), or ammonium (µM of ammonium = - 0.045*crayfish +1.108, *R^2^* = 0.082, P = 0.28). While these analyses focused on the average nutrient concentration, date-specific nutrient data also did not reveal associations with crayfish density (data not shown). Temperature and light did not perform substantially better as predictors of nutrient concentrations, with the exception of temperature being a substantial predictor of nitrate plus nitrate concentrations (next best ΔAICc = 3.6).

### Comparative Study

**Figure 3:**
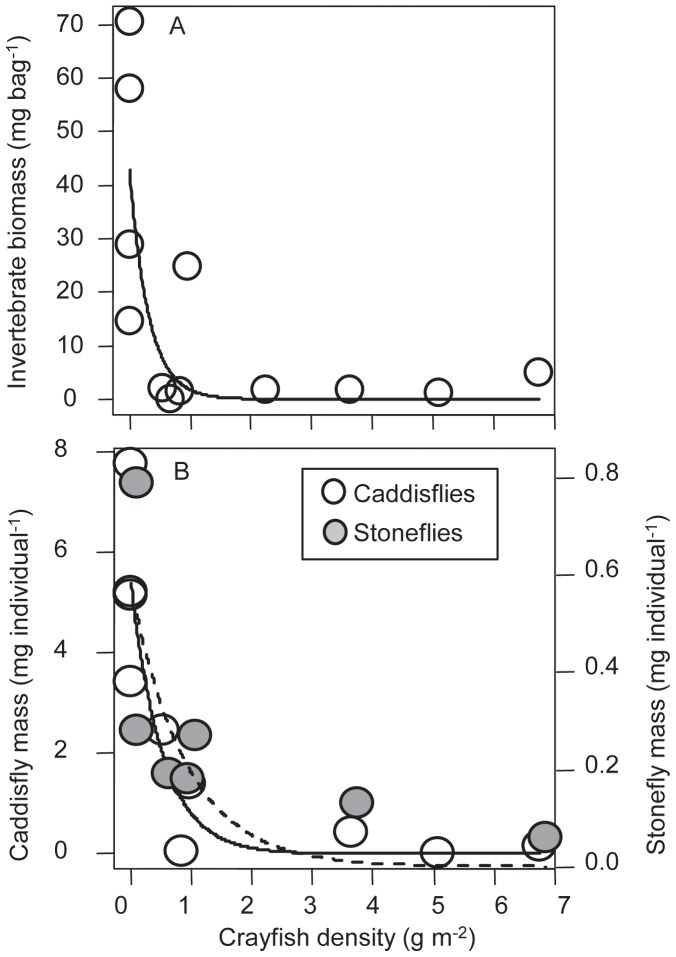
Invertebrate communities and the comparative study of crayfish. Each point represents the average for a study pool. (a) Invertebrate biomass (average biomass for coarse mesh leaf litter bags) as a function of crayfish density. Shown is the best fit exponential decay relationship (invertebrate biomass = 42.7 * exp (crayfish * −3.2)). (b). Average individual invertebrate mass as a function of crayfish density. White symbols with the solid line are caddisflies (Tricoptera), corresponding to the left y-axis and gray symbols with the dashed line correspond to stoneflies (Plecoptera), corresponding to the right y-axis. If pools did not contain any individuals, these points are not shown.

Comparisons of sites across an existing gradient of crayfish densities revealed different relationships among crayfish and stream communities and ecosystems. First, sites with crayfish had 9 times lower biomass of benthic invertebrates than sites without crayfish (Fig. 3a). The relationship between crayfish density and benthic invertebrate biomass was strongly non-linear, and an exponential model (invertebrate biomass = 42.7 * exp (crayfish * −3.2); Fig. 3a) explained much of the variance (R^2^ = 0.59) and was substantially better than the next best model (next best ΔAICc = 5.6; Electronic supplemental material Table S2). This pattern of a negative relationship between crayfish and benthic invertebrate biomass in the comparative study is qualitatively similar to experimental results. In contrast, variation in invertebrate abundance on litter bags was not explained well by crayfish density, temperature, nor light (*P*>0.4 for all; Electronic supplemental material Table S2). However, the average size of individual invertebrates was strongly associated was crayfish density. Specifically, caddisfly (order: Tricoptera) average size decreased from 5 mg individual^−1^ in pools with low crayfish densities to less than 0.5 mg individual^−1^ in pools with high crayfish densities (Fig. 3b). Similarly, stonefly (order: Plecoptera) average size decreased from 0.5 mg individual^−1^ in pools with low crayfish densities to less than 0.05 mg individual^−1^ in pools with high crayfish densities (Fig. 3b).

**Figure 4:**
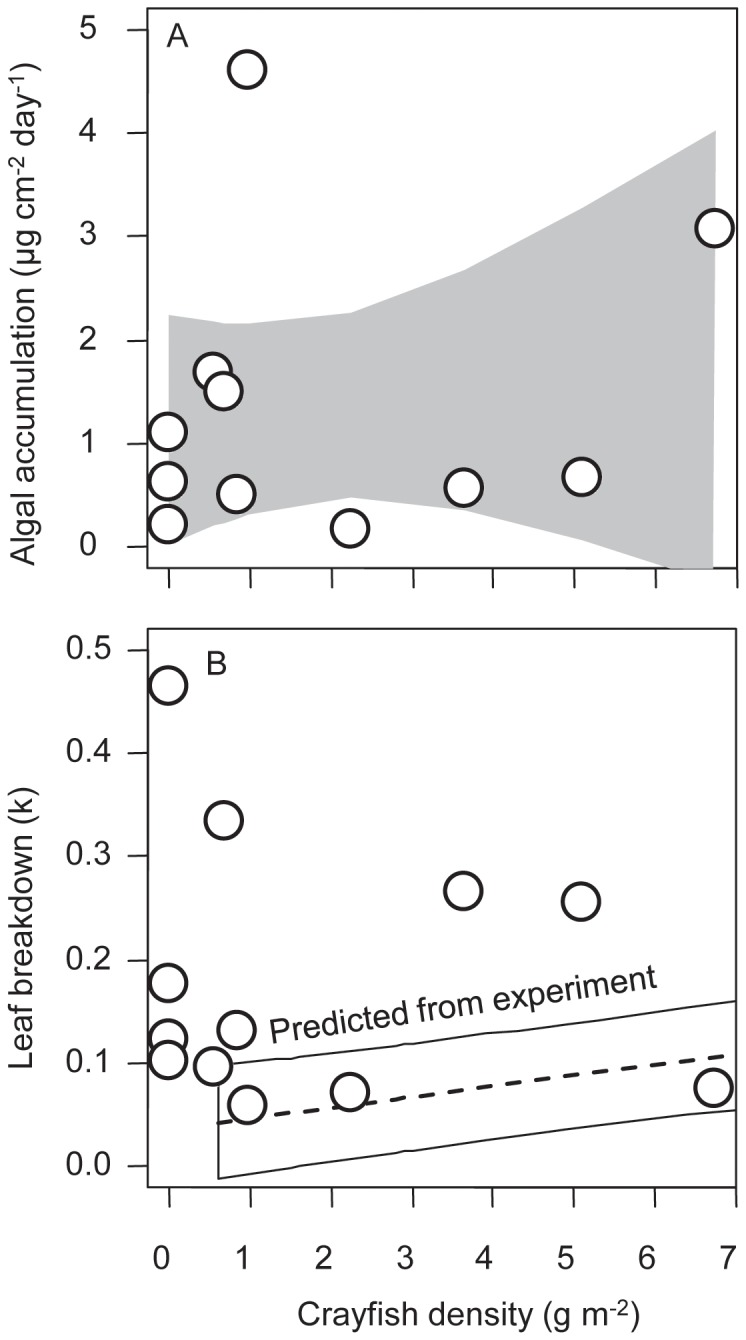
Ecosystem processes in the comparative study. Each point represents a different study pool. (a). Rate of algal accrual on elevated tiles. The gray polygon denotes the 95% confidence interval. (b). Rate of breakdown of leaves. Also shown in this panel in the open polygon and dashed line is the prediction interval and predicted line from the 2008 experimental relationship between leaf litter breakdown rate and crayfish.

Algal accrual rates in the comparative study were best explained by light levels. Specifically, cumulative light flux explained 66% and 42% of the variance in algal accrual for control and reference tiles, respectively (Electronic supplemental material Table S1). Light was substantially the best model, with the next best models having ΔAICc of 11.2 and 5.8 for control and elevated tiles, respectively (Electronic supplemental material Table S1). Crayfish density explained little of the variation in algal accrual (Fig. 4a; Electronic supplemental material Table S1).

Leaf litter breakdown rate was not significantly predicted by crayfish density (*R^2^ = *0.01, *P = *0.73; Fig. 4b), in contrast to the experimental results. Neither temperature nor light were significant predictors of breakdown rate (*P*>0.5). Based on the intercept of the estimated relationship between crayfish density and leaf litter breakdown from the 2008 experimental manipulation, we predicted leaf breakdown rate (k) to be 0.036±0.012 (mean ±1 SE) in the absence of crayfish (Fig. 4b). Instead, in pools without crayfish, k was 8 times higher than predicted. In addition, using the prediction interval from the 2008 experiment, the 2009 observations of high k at low crayfish densities were substantially higher than the range of predicted values.

### Feeding Trials

**Figure 5:**
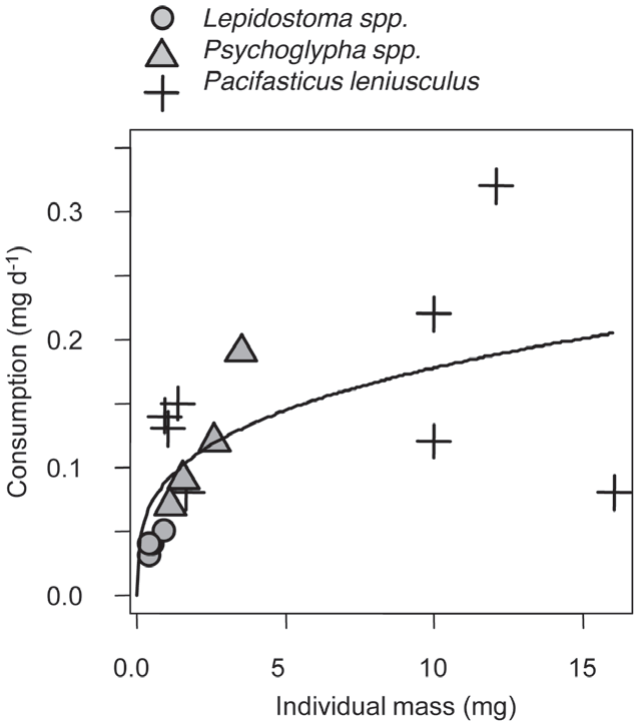
Consumption of leaf litter by three detritivores: the invasive signal crayfish (*P. leniusculus*), and the native caddisflies *Lepidostoma spp*. and *Psychoglypha* spp. (order: Tricoptera) in feeding trials. Individual mass is dry mass of the individual after the experiment. Consumption is the amount of leaves eaten, normalized by background loss rate (see Methods). Also shown is the best fit allometric relationship.

Both within and across taxonomic groups, larger individuals consumed more leaf litter (Fig. 5). This consumption generally followed an allometric scaling relationship such that consumption on a per-mass basis decreased with increasing size (Fig. 5; consumption (mg eaten d^−1^) = 0.009*biomass^0.29^). It appeared that both allometry across individuals and differences among taxa contribute to this pattern. For example, the average signal crayfish in the feeding trial was approximately 3.75 times the mass of the native *Psychoglypha* spp. caddisfly but only ate 1.3 times the amount of that caddisfly (Fig. 5).

## Discussion

We investigated the direct and indirect impacts of an invasive omnivore across temporal scales. We predicted that direct impacts would take primacy in the shorter term field experiment and that indirect impacts would dominate the longer term contrast of the field comparative study.

We thus used three approaches. First, we used a large-scale experiment to examine the short-term (three month) direct and indirect impacts of omnivorous crayfish on stream communities and ecosystems. Second, we implemented a comparative study that compared areas that were un-invaded and invaded 90 years ago to examine whether the patterns from the experiment scaled up to longer time frames. Third, we performed feeding trials to quantify consumption of leaf detritus by invasive crayfish and two potentially important native detritivores. In the short-term experiment, crayfish drove decreases in benthic invertebrates and detritus, likely driven by direct consumption, but were positively associated with algal accrual, likely mediated by an indirect effect (i.e., trophic cascade). The long-term comparative field study found that crayfish were negatively associated with benthic invertebrates, but not significantly related to detritus or algal accrual. These results support predictions of a temporally shifting balance of direct and indirect effects. Below we synthesize the results from these different studies for the different response variables.

**Figure 6:**
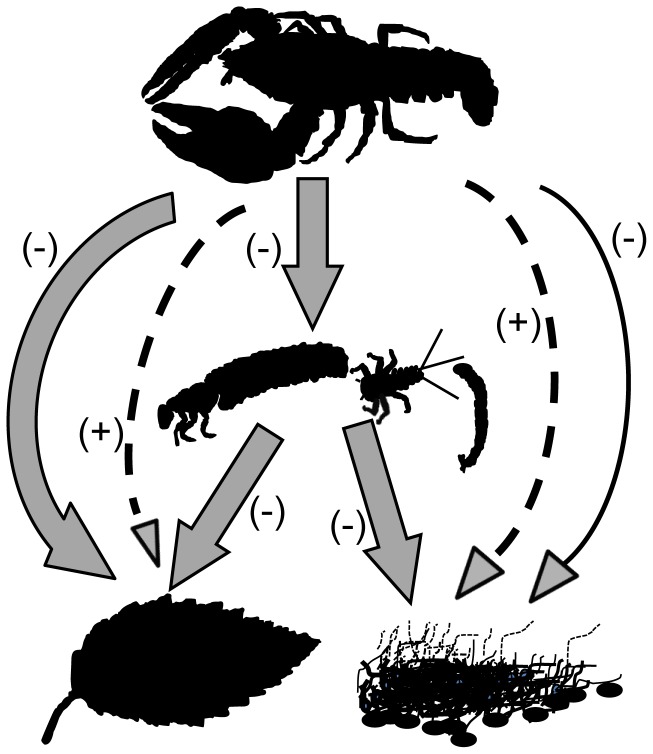
Schemata of the direct and indirect effects of crayfish (*P. leniusculus*). Direct effects are solid arrows, indirect effects are dashed arrows. The direction of the impact is shown in parenthesis.

Both the experiment and the comparative study found that non-crayfish benthic invertebrate biomass was dramatically reduced at higher densities of crayfish (Fig. 1, 3a). Chironomids, taxa with short generations that can be important grazers [48], were especially responsive to crayfish density in the experimental manipulation (see Fig. S1 in the Electronic supplemental material). Previous studies have also noted that crayfish can negatively impact chironomids either through bioturbation or consumption [49]–[51]. In the experiment, invertebrate abundance was also negatively associated with crayfish abundance. In contrast, in the comparative study where we quantified invertebrates through collecting them on leaf litter bags, crayfish were negatively associated with invertebrate biomass but not abundance (Electronic supplemental material Table S1, Table S2). Patterns of biomass in the comparative study were primarily driven by large caddisflies that appeared to be excluded by crayfish-large caddisflies such as *Psychoglypha* spp. and *Lepidostoma* spp. comprised 98% of total biomass of invertebrates in sites without crayfish (Fig. 3b). In contrast, in sites with crayfish, caddisflies composed an average of 34% of the biomass, the remainder was comprised of a mix of smaller stoneflies, mayflies, and chironomids. Thus, the experiment and comparative study found evidence of negative direct effects of crayfish on benthic invertebrates (Fig. 6). Previous studies have also noted that crayfish can exert strong negative direct effects on benthic invertebrates [52], [53].

The impacts of crayfish on algal accrual were variable. In the elevated tiles in the experiment, there was a significant positive association between crayfish density and algal accrual rate. This follows predictions based on a trophic cascade that appeared rapidly (<3 month). The observation that crayfish increased algal production on elevated tiles implies that native grazers were more mobile and were able to colonize the elevated tiles. These findings are similar to those of Charlebois and Lamberti [54], who combined an enclosure-exclosure experiment and a field survey across crayfish densities and found that crayfish were negatively associated with herbivorous invertebrates and positively associated with periphyton, indicating that the indirect effect of crayfish on algae via a trophic cascade was stronger than the direct effect of crayfish herbivory. Such trophic cascades have been widely described in aquatic systems [13]. While crayfish nutrient excretion could also be contributing to increasing primary production by increasing the availability of potentially limiting nutrients, an often overlooked potential mechanism by which consumers can increase primary productivity [55], [56], we did not observe significant relationships between crayfish density and nutrient concentrations in the experiment (Electronic supplemental material Table S1). There were no significant associations between crayfish and algal accrual on control tiles in the experiment (Electronic supplemental material Table S1). We speculate that environmental stochasticity, as well as the possibility that crayfish could more easily influence algal accrual on control tiles that were placed on stream sediments through multiple pathways such as bioturbation, fine sediment dynamics, and grazing pressure, contributed to this lack of a relationship. Crayfish also did not have strong associations with algal accrual in the comparative study. Instead, perhaps not surprisingly, light was the best predictor of algal accrual rate in the comparative study for both elevated and control tiles. It is intriguing that there was an apparent shift from biotic (crayfish) to abiotic (light) control of algal accrual from the experiment to the comparative study; however, the confidence intervals for the relationship between crayfish and algal accrual rate were large and overlapping between the experiment (Fig. 2a) and comparative study (Fig. 4a).

The impacts of crayfish on detritus were more complex. The experiment found that leaf litter breakdown rates were increased by crayfish (Fig. 2b); crayfish density explained 64% of the observed variation in leaf litter breakdown rate. Previous studies have also found that crayfish are important detritivores [37], [57]. Thus, crayfish have a strong negative direct effect on leaf litter via consumption (Fig. 6).

We hypothesize that crayfish also have a contrasting and strong positive indirect effect on leaf litter via consumption of native detritivores (Fig. 5). In contrast to the experiment, in the comparative study we did not observe significant differences in leaf litter breakdown rates between areas with and without crayfish. In fact, in the absence of crayfish we predicted leaf litter breakdown to be 8 times lower than we observed in the comparative study (Fig. 4b). In these sites without crayfish, the biomass and abundance of large bodied caddisfly detritivores was much higher, as noted above. Our feeding trials indicated that these large caddisflies as well as crayfish are effective detritivores (Fig. 5). Previous studies have noted that these caddisflies are important detritivores [58], have a 1+ year life cycle [48], and are vulnerable to crayfish predation [33]. Consistent with predictions from models of intraguild predation that the top consumer will generally exclude the intermediate consumer [9], crayfish appear to exclude these intermediate consumers (Fig. 3). These large-bodied and long-lived caddisflies were not observed at all in the experiment when crayfish were removed from sites; given this caddis’ generation times are on the order of 1+ years, their populations would not have had sufficient time to respond to the crayfish removal. We posit that during invasion, generalist crayfish replace the more specialized native detritivores (caddisflies), thereby leading to no systematic change in net ecosystem detrital breakdown. Thus, we hypothesize that crayfish have strong but contrasting direct negative and indirect positive effects on leaf litter (Fig. 6) that operate on different time scales.

This study adds to our understanding of the diverse pathways by which crayfish can influence aquatic ecosystems. Previous studies have observed that crayfish can be important detritivores [52], [59], herbivores [31], predators [36], [49], and ecosystem engineers [50], [60]. Thus, crayfish could have diverse and potentially interacting direct and indirect impacts on streams. Previous studies have noted the possibility that crayfish could have contrasting negative direct effects (i.e., consumption of detritus) and positive indirect effects on basal resources (i.e., consumption of other invertebrate detritivores) [49], [54]. To this point, studies have found that the net effect of crayfish on detritus has been negative, implying that direct consumption of detritus takes primacy over the indirect impacts of crayfish consumption of other detritivores. For example, Usio [49] used artificial stream channels to manipulate crayfish densities and found that crayfish were associated with decreased invertebrates and decreased detritus. As noted by Usio, this study was performed in New Zealand that has a paucity of other invertebrate detritivores, thereby reducing the potential importance of the cascading indirect impacts of crayfish on detritus. Other studies in regions with more diverse detritivore communities have also found that crayfish decrease benthic invertebrate size or abundance as well as detritus [50], [52]. These studies involved short-term manipulations of crayfish densities (similar in duration to our experimental study) using constructed stream channels. Our data suggests that crayfish are indeed important consumers of detritus, but, in contrast to previous findings, that they replace large-bodied native detritivores (order: Tricoptera) that are effective detritivores (Fig. 6). Given that it may take time for detritivore communities to develop after the removal of crayfish predators, especially the more efficient larger-bodied and longer-lived taxa (Fig. 5), it is possible that previous experiments were not long enough in duration for the full manifestation of indirect effects of crayfish.

The differences between the field experiment and comparative study illustrates the importance of considering temporal scale in interpreting results. Theory predicts that indirect effects will develop over longer time frames than direct effects, scaling to the generation time of the community components [22]. Density-mediated indirect effects entail the population-level response of the different food-web components, thus these indirect effects should occur no more rapidly than the direct effect and take as long as the generation time of the species in the indirect pathway [22]. While Menge [22] concluded that indirect effects often appeared simultaneously with direct effects in his review of rocky intertidal dynamics, temporal delays have been observed in experimental manipulations in other systems. For example, it took 20 years after the exclusion of kangaroo rats (*Dipodomys* spp) for its competitor, a large-bodied pocket mouse (*Chaetodipus baileyi*), to flourish and restore energy flow [61]. Examples of time lags in stream ecosystems include the 8 year time lag in the response of bryophytes to long-term fertilization [62] and the 1+ year time lag in the competitive release of benthic invertebrate populations from the disease-driven decimation of a dominant caddisfly [63]. Thus, short term experiments may predominantly show transient effects. The potential for time lags in propagation of indirect effects challenges our ability to scale up short-term experiments to understand how species alter long-term ecosystem processes [22], [24].

Reconciliation of the short-term experiment with the comparative study has several complicating factors. The comparative study and experiment were performed in different years; perhaps the years were characterized by different conditions driving the different results. However, both summers were qualitatively similar, characterized by low flows and no summer precipitation between July and September. In addition, we acknowledge that the sampling in 2009 was not randomly dispersed across the watershed (i.e., there was only one waterfall that blocked crayfish invasion). Furthermore, we do not have pre-invasion data (from 90+ years ago) for the comparative field study to ensure that crayfish were responsible for the observed patterns. We used a model selection approach with potentially strong environmental predictors such as light in our analyses in order to address this possibility. While large-scale, long-term, and well-replicated experiments are the obvious ideal [61], [64], studies such as ours that reconcile results from comparative and manipulative studies may provide one method of integrating both process and long-term patterns. Given the logistic challenges of long-term manipulations, it is not surprising that studies continue to use short-term manipulations to examine the impacts of species on ecosystem processes. However, such field experiments may only quantify transient dynamics as indirect effects may take generations for manifestation [22], [24]. Scaling issues remain a challenge for ecology [65], [66]. For example, had we based our results on just the 3 month experimental manipulation, we would have concluded that direct effects of crayfish dominate detrital dynamics, even though this effect may not be balanced by the opposing indirect effect over time (Fig. 6). Thus, the impacts of this invasive omnivore represent a temporally-shifting interplay between direct and indirect effects that can control both detritus and primary productivity.

## Supporting Information

Figure S1 **Biomass of key invertebrate groups as a function of crayfish density from the 2008 density manipulation.** Note y-axis data is log +1 transformed. Each points represents the invertebrate abundance of a given study pool from surber samplers, as a function of manipulated crayfish density. The lines in represent the best fit linear model that were significant at the *P* = 0.05 level.
(DOCX)

Table S1 **Results of model comparison for the 2008 experiment.** ΔAICc is used to compare contrasting models; lower ΔAICc indicate a better model. Models that were substantially better, as evidenced by a ΔAICc of at least 2.0 of the next best model, are bolded. All models were basic linear models on either untransformed or transformed data. *P*-values refer to the significance of the model against a null model of no relationship.
(DOCX)

Table S2 **Results of model comparison for the 2009 comparative study.** Unless otherwise noted, all models are basic linear models.
(DOCX)
