# Supplementary material for: Trophic Tangles through Time? Opposing Direct and Indirect Effects of an Invasive Omnivore on Stream Ecosystem Processes
Source: PLoS One. 2012 Nov 27;7(11):e50687. doi: 10.1371/journal.pone.0050687 (PMC3507779; doi:10.1371/journal.pone.0050687)
Supplement: Figure S1 — Biomass of key invertebrate groups as a function of crayfish density from the 2008 density manipulation. Note y-axis data is log +1 transformed. Each points represents the invertebrate abundance of a given study pool from surber samplers, as a function of manipulated crayfish density. The lines in represent the best fit linear model that were significant at the P = 0.05 level. (DOCX) [file pone.0050687.s001.docx]

**Electronic Supplemental Material Fig. S1.** Biomass of key invertebrate groups as a function of crayfish density from the 2008 density manipulation. Note y-axis data is log + 1 transformed. Each points represents the invertebrate abundance of a given study pool from surber samplers, as a function of manipulated crayfish density. The lines in represent the best fit linear model that were significant at the *P* = 0.05 level.
