# Supplementary material for: Trophic Tangles through Time? Opposing Direct and Indirect Effects of an Invasive Omnivore on Stream Ecosystem Processes
Source: PLoS One. 2012 Nov 27;7(11):e50687. doi: 10.1371/journal.pone.0050687 (PMC3507779; doi:10.1371/journal.pone.0050687)
Supplement: Table S1 — Results of model comparison for the 2008 experiment. ΔAICc is used to compare contrasting models; lower ΔAICc indicate a better model. Models that were substantially better, as evidenced by a ΔAICc of at least 2.0 of the next best model, are bolded. All models were basic linear models on either untransformed or transformed data. P-values refer to the significance of the model against a null model of no relationship. (DOCX) [file pone.0050687.s002.docx]

**Electronic supplemental material Table S1.** Results of model comparison for the 2008 experiment. ΔAICc is used to compare contrasting models, lower ΔAICc indicate a better model. Models that were substantially better, as evidenced by a ΔAICc of at least 2.0 of the next best model, are bolded. All models were basic linear models on either untransformed or transformed data. *P*-values refer to the significance of the model against a null model of no relationship.

Response Variable Factor ΔAICc *R^2^* *P*-value

**Invert mass^1^ Crayfish 0 0.33 0.02**

Invert mass^1^ Temperature 6.4 0.002 0.88

Invert mass^1^ Light 6.2 0.093 0.72

**Invert abundance^1^ Crayfish 0 0.59 0.005**

Invert abundance^1^ Temperature 11.6 0.15 0.13

Invert abundance^1^ Light 14.3 0.002 0.86

Algae--control Crayfish 0.0 0.08 0.29

Algae--control Temperature 1.3 0.002 0.88

Algae--control Light 1.1 0.013 0.67

**Algae—elevated Crayfish 0.0 0.29 0.03**

Algae—elevated Temperature 5.5 0.00 0.99

Algae—elevated Light 4.6 0.05 0.40

**K Crayfish 0 0.64 0.0002**

K Temperature 16.4 0.003 0.85

K Light 16.0 0.03 0.54

**Carbon^1^ Crayfish 0 0.39 0.02**

Carbon^1^ Temperature 4.6 0.09 0.26

Carbon^1^ Light 5.5 0.035 0.488

Nitrate and nitrite^2^ Crayfish 3.6 0.08 0.27

**Nitrate and nitrite**^2^ **Temperature 0.0 0.27 0.04**

Nitrate and nitrite^2^ Light 5.0 0.000 0.93

Phosphorus^2^ Crayfish 0.7 0.002 0.85

Phosphorus^2^ Temperature 0.0 0.049 0.41

Phosphorus^2^ Light 0.1 0.042 0.45

Ammonium^2^ Crayfish 0.2 0.082 0.28

Ammonium^2^ Temperature 1.2 0.020 0.61

Ammonium^2^ Light 0.0 0.093 0.25

^1^These variables were log transformed prior to analysis

^2^These variables represent averages of three measurements over the course of the experiment. Running these analyses with date-specific values had similar lack of strong predictor models (analyses not shown).
