# Supplementary material for: Trophic Tangles through Time? Opposing Direct and Indirect Effects of an Invasive Omnivore on Stream Ecosystem Processes
Source: PLoS One. 2012 Nov 27;7(11):e50687. doi: 10.1371/journal.pone.0050687 (PMC3507779; doi:10.1371/journal.pone.0050687)
Supplement: Table S2 — Results of model comparison for the 2009 comparative study. Unless otherwise noted, all models are basic linear models. (DOCX) [file pone.0050687.s003.docx]

**Electronic supplemental material Table S2.** Results of model comparison for the 2009 comparative study. Unless otherwise noted, all models basic linear models. For a more thorough description of AICc, see Electronic supplemental material Table S1.

Response Variable Factor ΔAICc *R^2^* *P*-value

K Crayfish 0.3 0.012 0.73

K Temperature 0.0 0.04 0.53

K Light 0.4 0.003 0.86

Algae—Control Crayfish 12.9 0.02 0.69

Algae—Control Temperature 11.2 0.15 0.22

**Algae—Control Light 0 0.66 0.001**

Algae—Elevated Crayfish 6.3 0.034 0.57

Algae—Elevated Temperature 5.8 0.077 0.38

**Algae—Elevated Light 0.0 0.429 0.02**

Invert abundance Crayfish 0.2 0.051 0.48

Invert abundance Temperature 0.6 0.02 0.66

Invert abundance Light 0.0 0.069 0.41

**Invert biomass Crayfish (exp)^1^0.0 0.59^2^ 0.0003**

Invert biomass Crayfish 7.6 0.23 0.11

Invert biomass Temperature 5.6 0.34 0.04

Invert biomass Light 10.4 0.026 0.62

^1^This model was *invertebrate biomass ~ a*exp(crayfish*z)*, where *a* and *z* are estimated parameters.

^2^Given that this was a nonlinear model, *R^2^* was approximated as the pseudo-*R^2^* based on 1-residual sum of squares/total sum of squares.
